# Draft Genome Sequence of *Corynebacterium kefirresidentii* SB, Isolated from Kefir

**DOI:** 10.1128/genomeA.00877-17

**Published:** 2017-09-14

**Authors:** Sonja Blasche, Yongkyu Kim, Kiran R. Patil

**Affiliations:** Structural and Computational Biology Unit, European Molecular Biology Laboratory, Heidelberg, Germany

## Abstract

The genus *Corynebacterium* includes Gram-positive species with a high G+C content. We report here a novel species, *Corynebacterium kefirresidentii* SB, isolated from kefir grains collected in Germany. Its draft genome sequence was remarkably dissimilar (average nucleotide identity, 76.54%) to those of other *Corynebacterium* spp., confirming that this is a unique novel species.

## GENOME ANNOUNCEMENT

Kefir grains are traditionally used as starters in the production of kefir fermented milk. They contain a stable consortium composed of 40 to 50 different species, both prokaryotes and eukaryotes (1). Among the bacterial species, acetic acid bacteria and lactic acid bacteria, including *Lactobacillus*, *Lactococcus*, and *Leuconostoc* spp., are dominant and play important roles in milk fermentation and kefir flavor (2). So far, the functional role of other low-abundance species remains unknown. Here, we isolated *Corynebacterium kefirresidentii* SB from a kefir grain collected from a private source in Offenberg, Germany.

*C. kefirresidentii* SB (internal stock no. 285) was isolated from ground kefir grains (internal name, Kefir OG2), plated in serial dilutions on tomato juice agar (TJA), and grown for 2 days at 30°C. The isolated clone was suspended in TES buffer (25 mM Tris, 10 mM EDTA, and 10 mM sucrose) and digested with lysozyme for 30 min at 37°C, followed by two bead-beating steps (30 s, 6.5 m/s) with an intermediate break of 1 min. Cells were lysed by addition of 3% SDS for 5 min at room temperature, followed by proteinase K (0.2 mg/ml final concentration) digestion for 30 min at 37°C. Digested protein was precipitated for 15 min on ice in 1 M potassium acetate (final concentration) and centrifuged for 15 min at 4°C. The supernatant was purified using standard phenol-chloroform extraction. DNA was precipitated by the addition of 2 volumes of ice-cold isopropanol and washed with 70% ethanol at 4°C.

DNA library creation and sequencing were done at the EMBL Genomics Core Facility (Heidelberg, Germany). It was sequenced on the Illumina HiSeq 2000 with 100-bp paired-end reads. A total of 3,941,660 read pairs were generated. The raw reads were assessed for the quality-based trimming and filtering by PRINSEQ (3). The qualifying read pairs were assembled using SPAdes 3.10.0 (4). Contigs shorter than 500 bp were discarded. The total number of contigs in the scaffold is 81, with the largest contig of 579,517 bp and an *N*_50_ value of 94,942 bp. The draft genome size is 2,522,639 bp, with a G+C content of 58.47%.

The NCBI Prokaryotic Genome Annotation Pipeline (PGAP) identified the genome as harboring 2,642 protein-coding genes, 51 tRNA genes, and 4 rRNA genes (2, 1, and 1, respectively, for 5S, 16S, and 23S rRNA) (5).

### Accession number(s).

The NCBI accession number of this whole-genome sequence is NGUZ00000000.
